# Usefulness of combining electrocardiogram and echocardiography findings and brain natriuretic peptide in early detection of cardiac amyloidosis in subjects with transthyretin gene mutation

**DOI:** 10.1186/1750-1172-10-S1-P34

**Published:** 2015-11-02

**Authors:** Gianluca Di Bella, Fabio Minutoli, Anna Mazzeo, Matteo Cssale, Claudia Stancanelli, Scipione Carerj, Sergio Baldari, Giuseppe Vita

**Affiliations:** 1 University of Messina, Clinical and experimental medicine department, 98100, Messina, Italy; 2 University of Messina, Department of Biomedical Sciences and of Morphologic and Functional Images, 98100, Messina, Italy; 3 University of Messina, Department of Neurosciences, 98100, Messina, Italy

## 

Early non-invasive identification of cardiac amyloidosis (CA) is of growing clinical importance. Low voltage on electrocardiogram (ECG), increased left ventricular (LV) septal thickness (ST) and global longitudinal strain (GLS) on echocardiography, and elevated brain natriuretic peptides (BNP) are used as surrogates of CA. Thirty-five patients (50 ± 14 years, 22 females) underwent an ECG to analyze low-voltage QRS (<15 mV) pathological Q-waves, poor R-wave progression, ST-T abnormalities - and left bundle branch block. An ECG was considered abnormal if at least one ECG alteration was present. Echocardiography was used to analyze LVST, E/E’ and GLS. All participants also had BNP blood testing. 99mTc-DPD scintigraphy assumed as a reference method showed CA in 18 patients (51%, CA group) and no accumulation in 17 patients (no CA group). In descending order of accuracy, LVST >14 mm, E/E’ >6.6, GLS <14.1, BNP >129 pg/ml, and an overall abnormal ECG showed good capability to distinguish patients with and without CA. All these parameters were predictors of CA in univariate analysis while low-voltage QRS showed the worst performance. LVST >14 mm (p = 0.002) was the best independent predictor of CA, achieving sensitivity of 78% and accuracy of 89%. However, a LVST >14 mm (p = 0.005) plus an abnormal ECG (p = 0.03) show together a higher sensitivity, equal to 89%, in identifying CA. An integrated evaluation of ECG and echocardiography is a sensitive and low-cost technical approach to identify CA in patients with transthyretin gene mutation.

